# Supplementary material for: Polyaniline-Derived N-Doped Ordered Mesoporous Carbon Thin Films: Efficient Catalysts towards Oxygen Reduction Reaction
Source: Polymers (Basel). 2020 Oct 16;12(10):2382. doi: 10.3390/polym12102382 (PMC7602833; doi:10.3390/polym12102382)
Supplement: Supplementary file 1 [file polymers-12-02382-s001.pdf]

Supporting Information

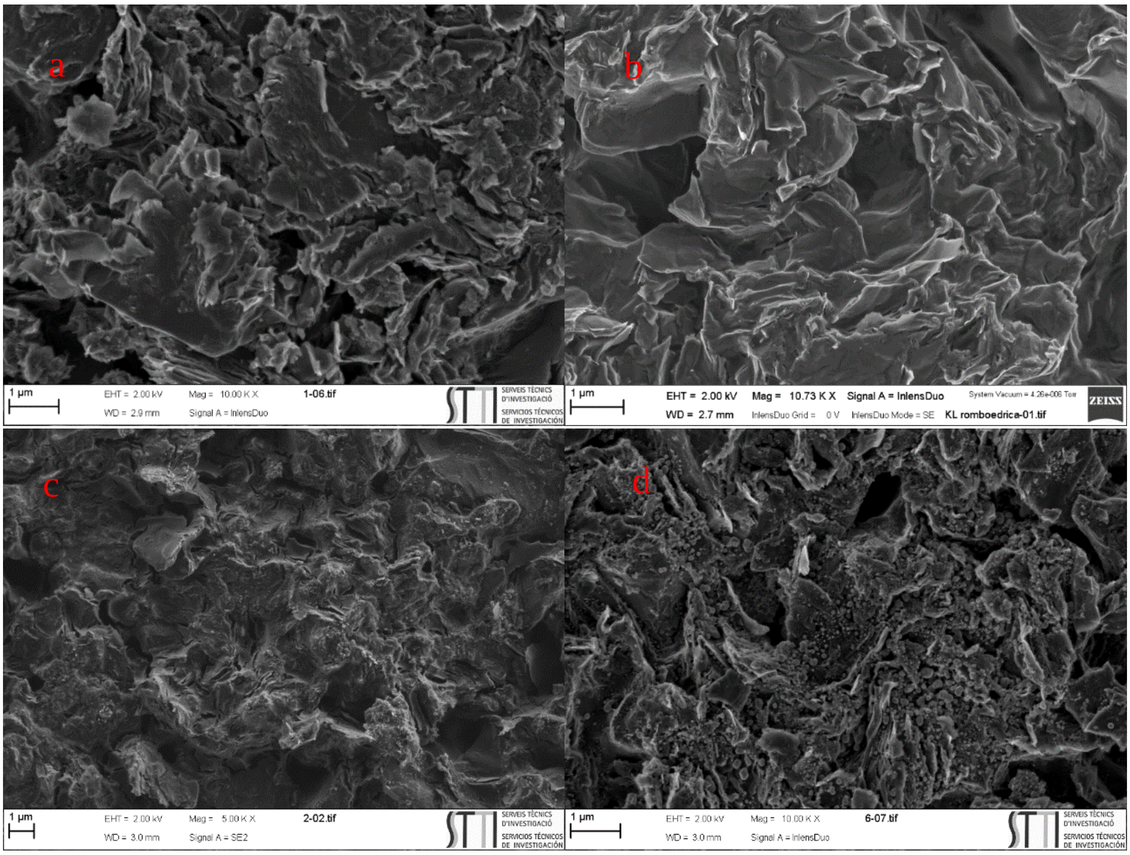

Figure S1: FESEM images of (a) G, (b) G/Si, (c) G/Ti and (d) G/Ti-900.

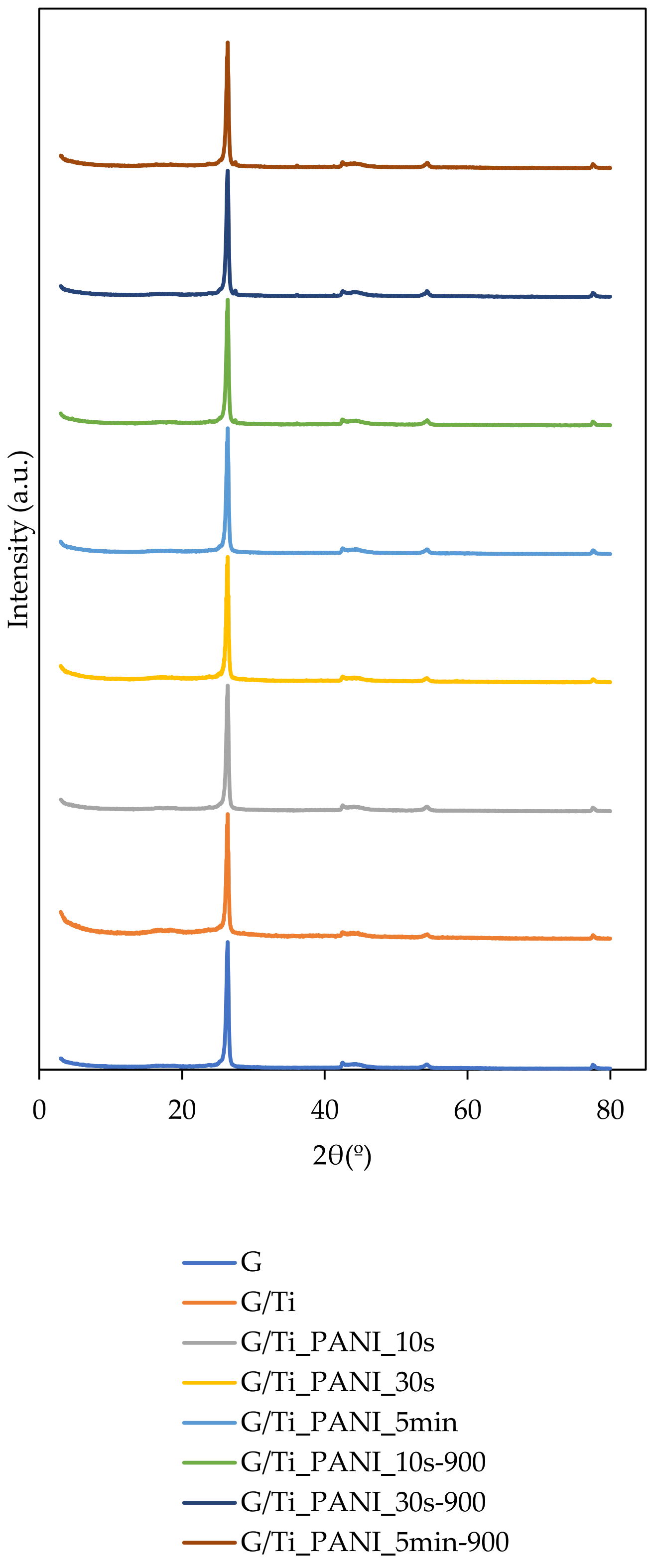

Figure S2: XRD patterns of all Titania composites.

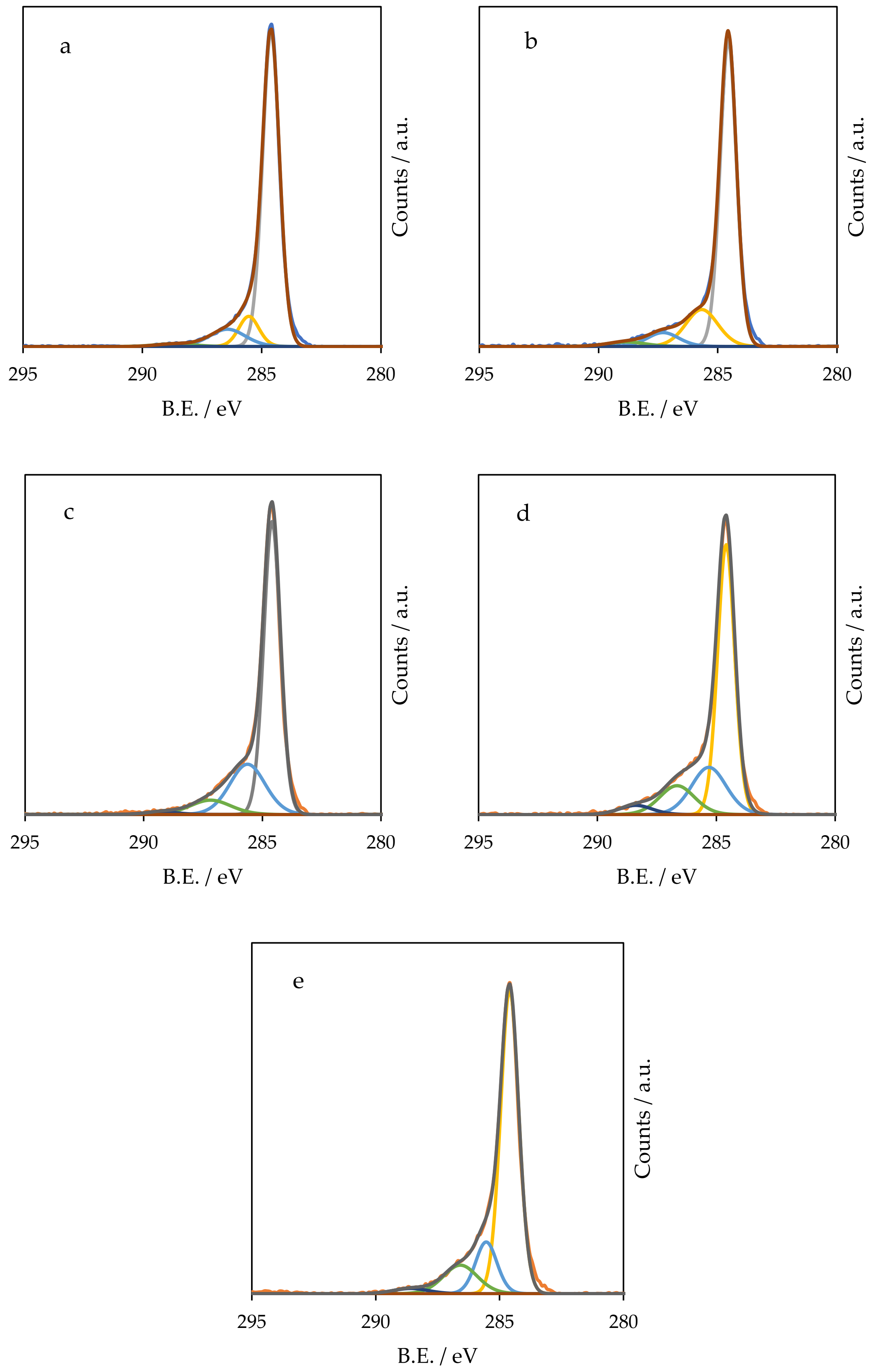

Figure S3: C1s spectra of (a) G, (b) G/Si, (c) G/Si_PANI_10s, (d) G/Si_PANI_30s, (e) G/Si_PANI_5min.

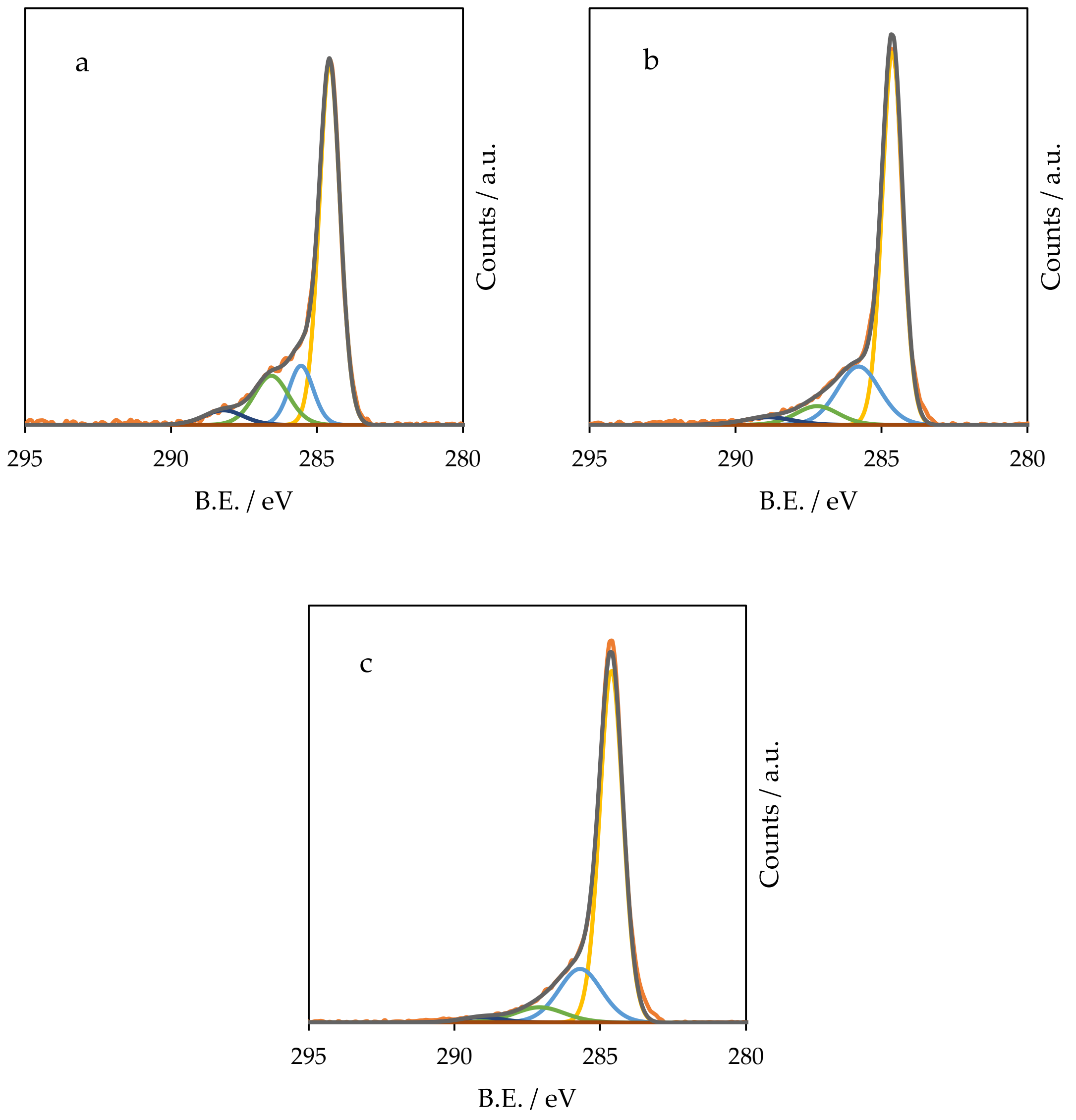

Figure S4: C1s spectra of (a) G/Si_PANI_10s-900, (b) G/Si_PANI_30s-900 and (c) G/Si_PANI_5min-900.

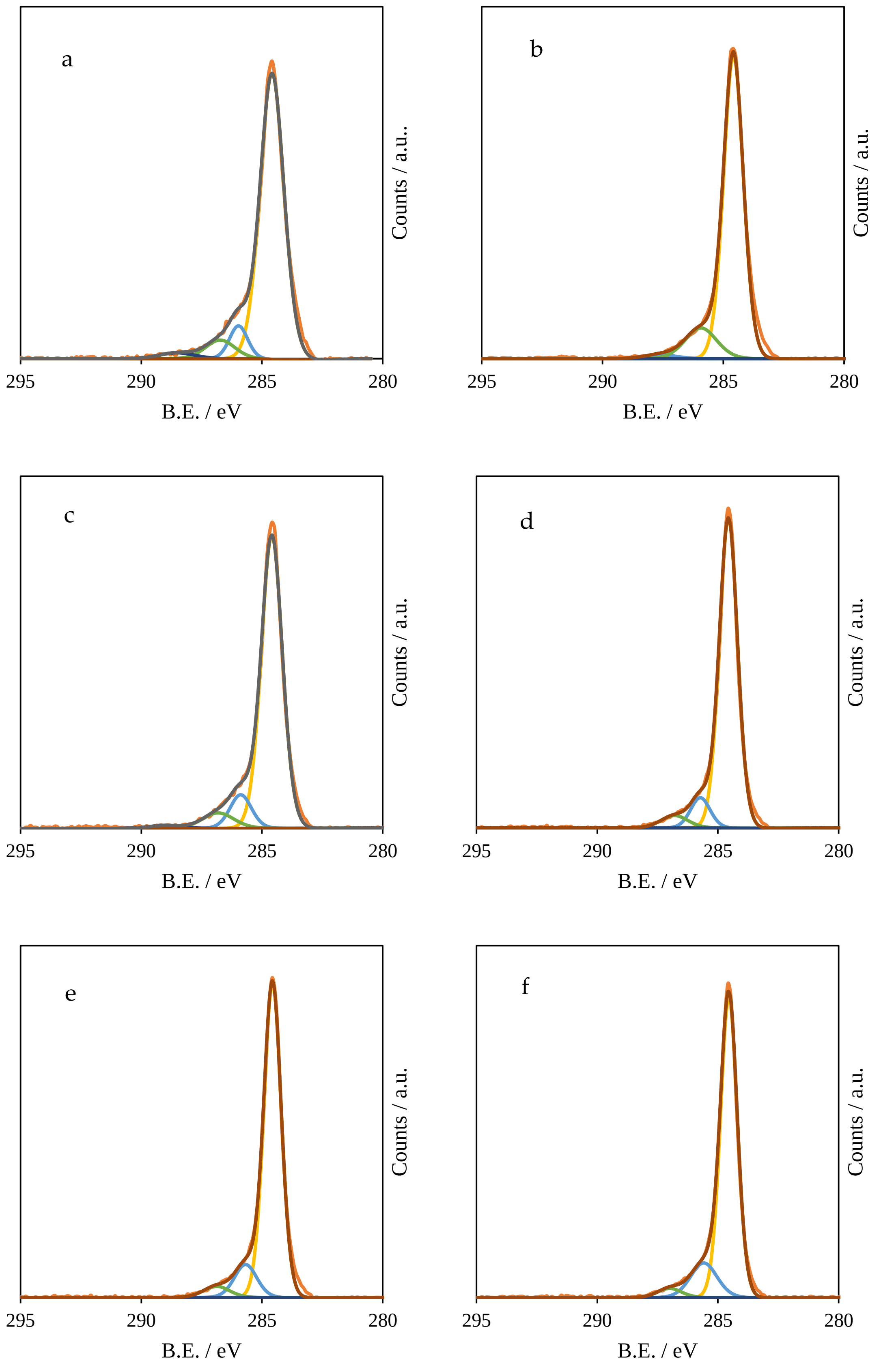

Figure S5: C1s spectra of (a) G/Ti_PANI_10s, (b) G/Ti_PANI_30s, (c) G/Ti_PANI_5min, (d) G/Ti_PANI_10s-900, (e) G/Ti_PANI_30s-900 and (f) G/Ti_PANI_5min-900.

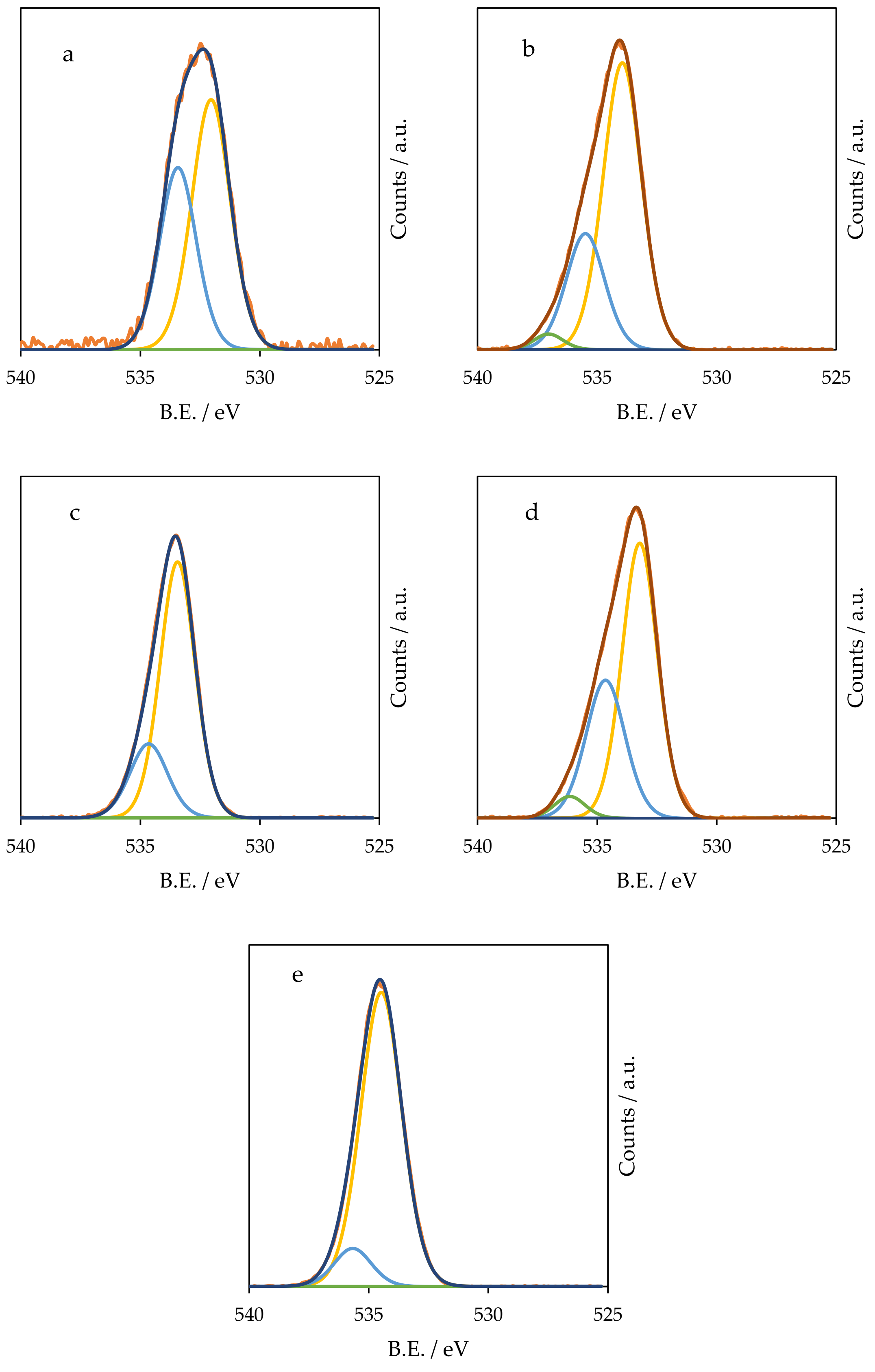

Figure S6: O1s spectra of (a) G, (b) G/Si, (c) G/Si_PANI_10s, (d) G/Si_PANI_30s and (e) G/Si_PANI_5min.

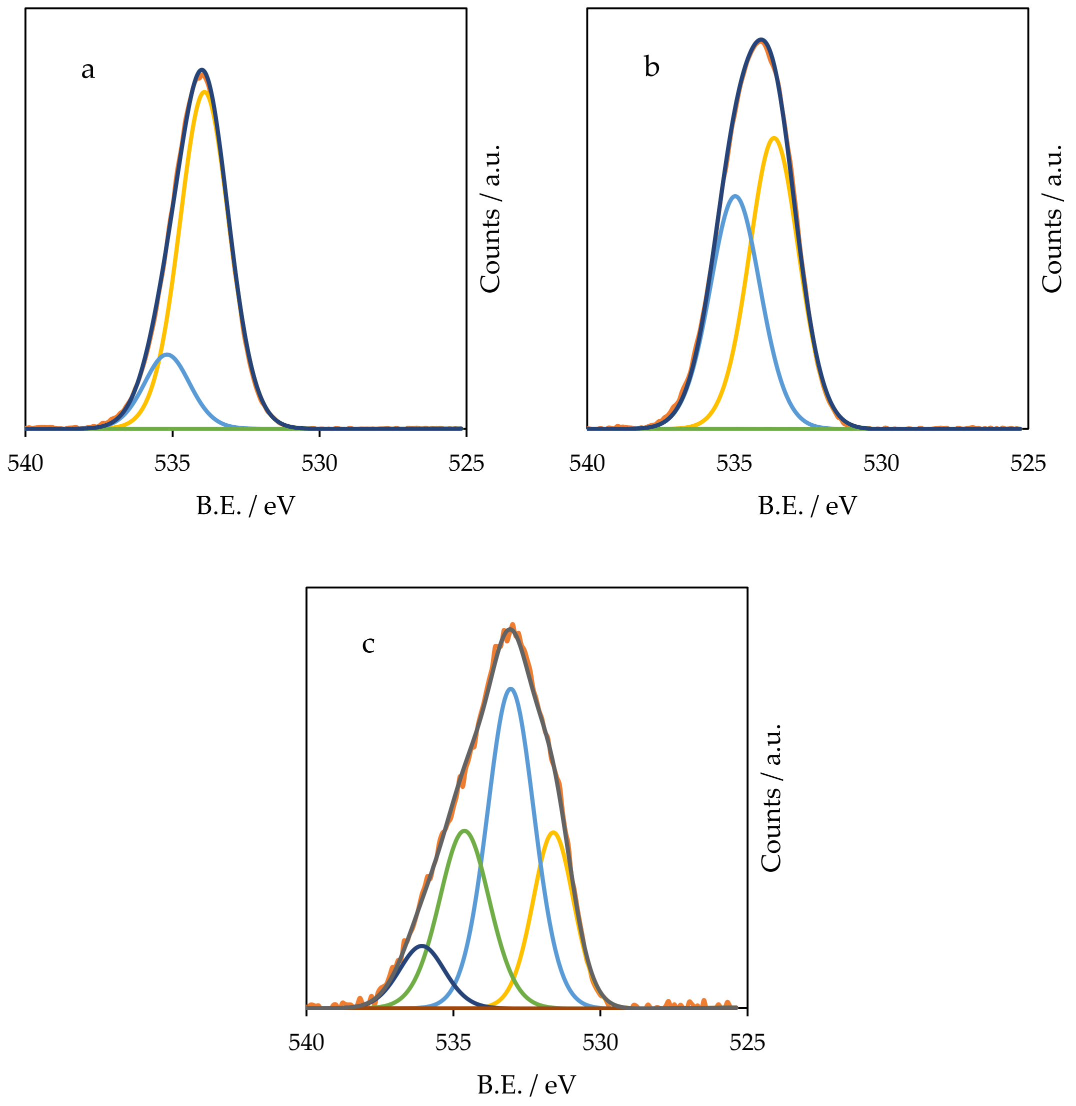

Figure S7: O1s spectra of (a) G/Si_PANI_10s-900, (b) G/Si_PANI_30s-900 and (c) G/Si_PANI_5min-900.

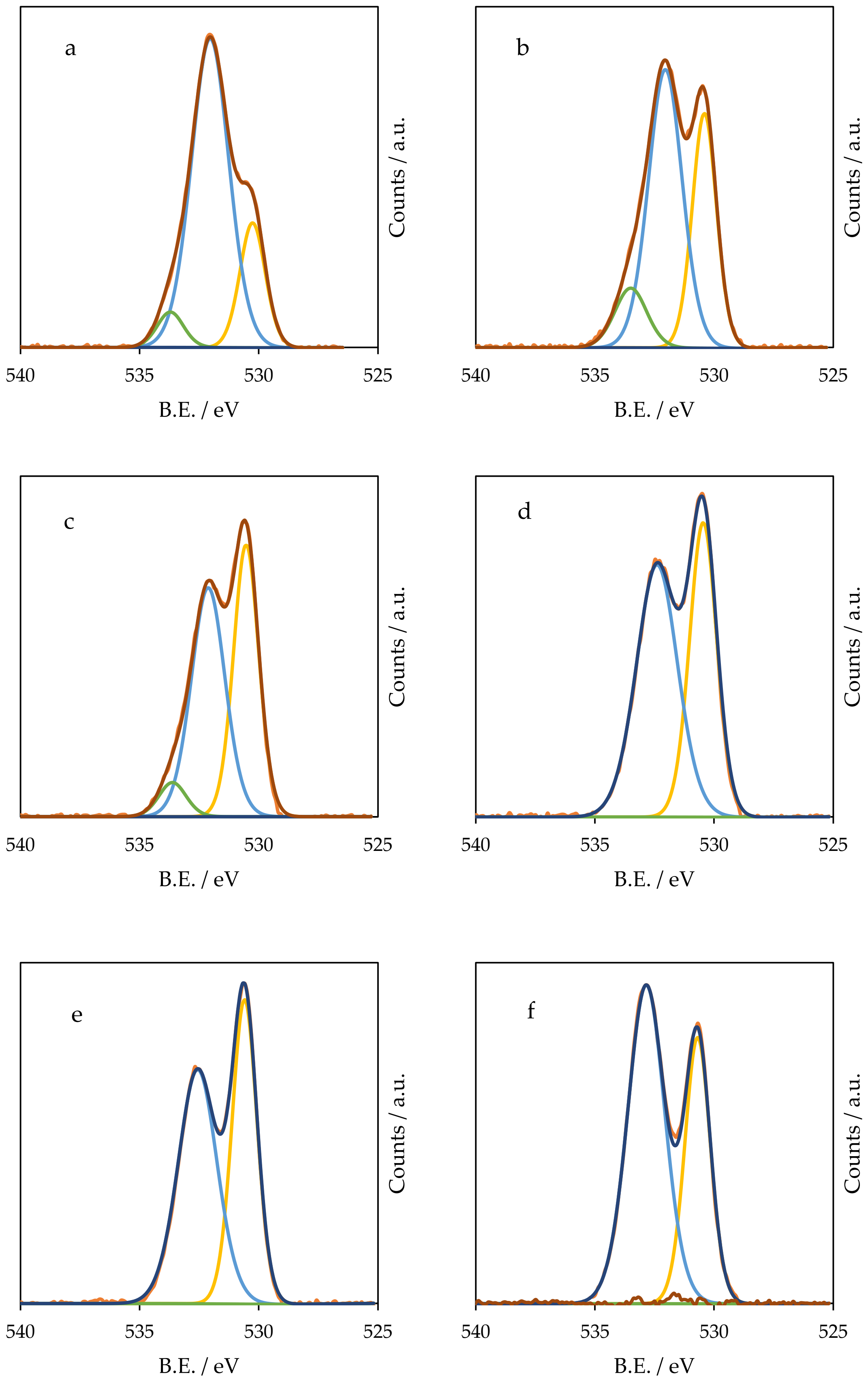

Figure S8: O1s spectra of (a) G/Si_PANI_10s, (b) G/Si_PANI_30s, (c) G/Si_PANI_5min, (d) G/Si_PANI_10s-900, (e) G/Si_PANI_30s-900 and (f) G/Si_PANI_5min_900.

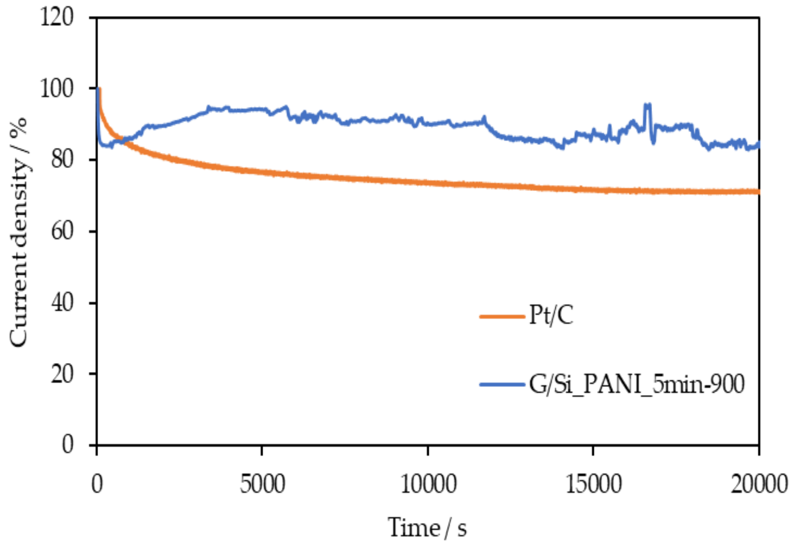

Figure S9: Chronoamperometric test of G/Si_PANI_5min-900 (blue line) and Pt/C (orange line). E = 0.65 V vs RHE.

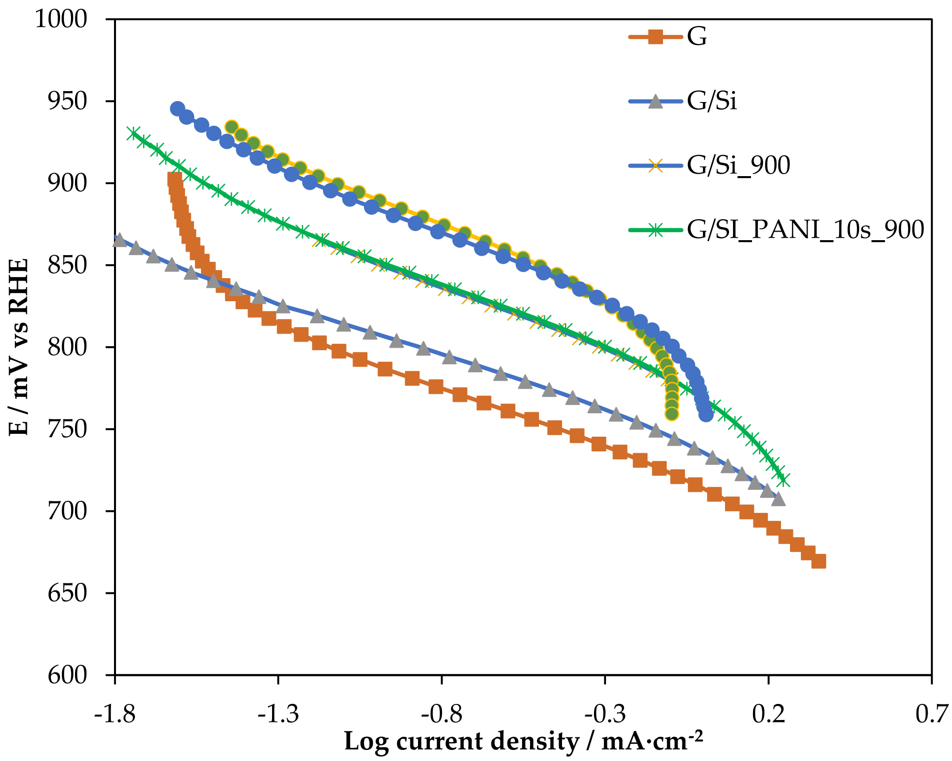

Figure S10: Tafel plots of the ORR of G/Ti samples in O_2-saturated 0.1 M KOH solution at 25ºC, 5 mV·s^{-1}.

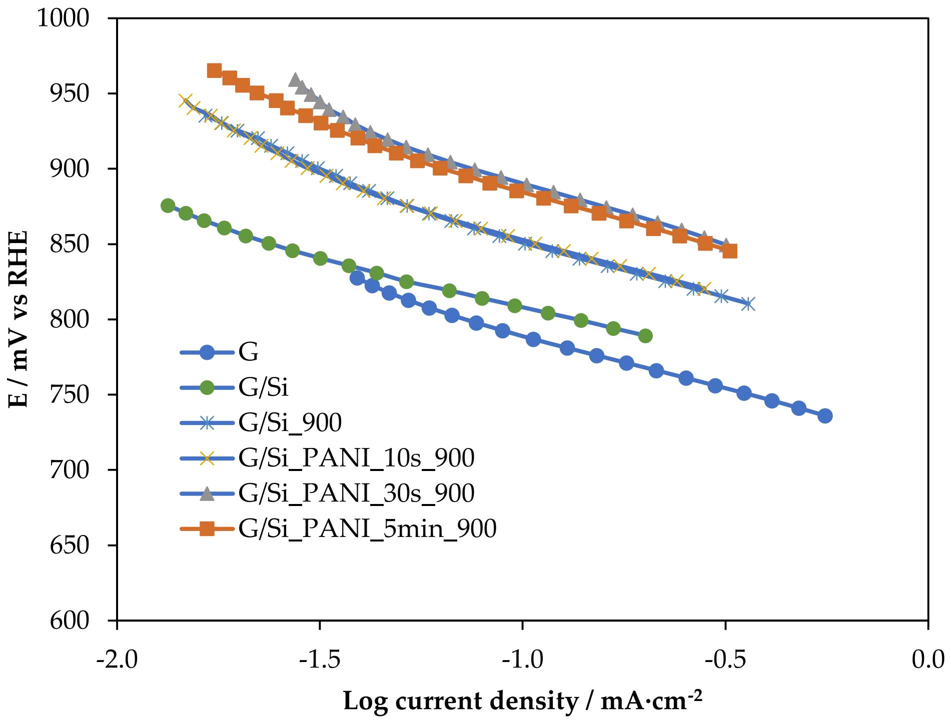

Figure S11: Tafel plots of the ORR of G/Si samples in O_2-saturated 0.1 M KOH solution at 25ºC, 5 $mV \cdot s^{-1}$.
